# Looking for *Lepiotapsalion* Huijser & Vellinga (Agaricales, Agaricaceae)

**DOI:** 10.3897/mycokeys.52.34021

**Published:** 2019-05-09

**Authors:** Alfredo Vizzini, Alessia Tatti, Henk A. Huijser, Jun F. Liang, Enrico Ercole

**Affiliations:** 1 Department of Life Sciences and Systems Biology, University of Torino, Viale P.A. Mattioli 25, I-10125, Torino, Italy University of Torino Torino Italy; 2 Institute for Sustainable Plant Protection (IPSP)-CNR, Viale P.A. Mattioli 25, I-10125, Torino, Italy Institute for Sustainable Plant Protection Torino Italy; 3 Department of Environmental and Life Science, Section Botany, University of Cagliari, Viale S. Ignazio 1, I-09123, Cagliari, Italy University of Cagliari Cagliari Italy; 4 Frederikstraat 6, 5671 XH Nuenen, The Netherlands Unaffiliated Nuenen Netherlands; 5 Research Institute of Tropical Forestry, Chinese Academy of Forestry, Guangzhou, 510520, China Research Institute of Tropical Forestry, Chinese Academy of Forestry Guangzhou China

**Keywords:** Agaricomycetes, Basidiomycota, cryptic species, hymeniform pileus covering, taxonomy

## Abstract

*Lepiotapsalion* is fully described based on a recent collection from Sardinia (Italy) and the holotype. NrITS- and nrLSU-based phylogeny demonstrates that sequences deposited in GenBank as “*L.psalion*” and generated from two Dutch and one Chinese collections are not conspecific with the holotype and represent two distinct, undescribed species. These species are here proposed as *Lepiotarecondita* sp. nov. and *Lepiotasinorecondita* ad int.

## Introduction

Recent molecular analyses have indicated that the genus *Lepiota* (Pers.) Gray is a paraphyletic assemblage that is monophyletic only if it is considered together with species of *Cystolepiota* Singer, *Echinoderma* (Locq. ex Bon) Bon, *Melanophyllum* Velen., and *Pulverolepiota* Bon (Johnson 1999; Vellinga 2003, 2004; Vellinga et al. 2011). Consequently, according to the modern concept of Vellinga (2003, 2004), the genus *Lepiota* s.l. includes the pale-spored members of the Agaricaceae Chevall., which are circumscribed by having non-metachromatic, dextrinoid, and usually binucleate spores, cheilocystidia usually present, pleurocystidia absent, a regular hymenophoral trama, and clamp-connections usually present. The structure of the pileus covering has been shown to be a key character to divide the genus into operative, morphology-based sections (Vellinga and Huijser 1999; Vellinga 2001, 2003, 2010).

Species of *Lepiota* with a hymeniform pileus covering were distributed by Bon (1993) over three different sections, *Cristatae* (Kühner ex Wasser) Bon, *Integrellae* (Kühner ex Bon) Bon and *Lilaceae* Bon, based mainly on different spore shapes (either ellipsoid or spurred) and spore nuclear number (mononucleate vs binucleate); all species were included by Vellinga and Huijser (1999) and Vellinga (2001) in an emended large sectionLilaceae.

According to recent molecular analyses, the species with a hymeniform pileus covering do not form a monophyletic lineage (Vellinga 2003, 2004, 2010; Vizzini et al. 2014a, b; Justo et al. 2015; Qasim et al. 2015; Hosen et al. 2016), even though most of them (with different spore shapes and nuclear number) fall in a clade (named clade 3 by Vellinga 2003) which also includes taxa as *L.albogranulosa* T. Qasim & A.N. Khalid, *L.cystophoroides* Joss. & Riousset, *L.luteophylla* Sundb., and *L.scaberula* Vellinga with a hymeniderm giving rise to loose globose elements (a transition between hymeniderm and epithelium, Vellinga 1988).

During a 3-year survey of macrofungi in the Botanical Garden of Cagliari (Sardinia, Italy), a collection of a *Lepiota* with a hymeniform pileus covering was recorded. It showed striking morphological affinities with *L.psalion* Huijser & Vellinga. The present paper fully describes this collection using morphological features and molecular data, and infers, through sequencing of the holotype, the phylogenetic placement of *L.psalion*. Additionally, two morphologically allied taxa, *Lepiotarecondita* sp. nov. and *L.sinorecondita* ad int. are described.

## Materials and methods

### Morphology

Macroscopic description was based on detailed field notes of fresh basidiomes. Colour terms in capital letters (e.g., Pale Cinnamon-Pink, Plate XXIX) are those of Ridgway (1912). HTML alphanumeric colour codes (https://html-color-codes.info/) were obtained using GIMP (GNU Image Manipulation Program, https://www.gimp.org/) with the “Color Picker” tool on photographs taken in natural light of fresh basidiomes. Micromorphological features were observed on dried material; sections were rehydrated in water or 5% KOH and mounted separately in ammoniacal Congo Red, Cotton Blue, Cresyl Blue, and Melzer’s reagent. Measurements of the microscopic features of *Lepiotapsalion* and *L.recondita* were made by photographing all the elements occurring in the visual field of an Optika B-383 PLi light microscope. Measurements were performed using the Piximètre 5.9 R 1530 software (http://ach.log.free.fr/Piximetre/) at 1000× magnification. The microphotographs were taken by an Optikam B5, 5 MP× camera.

When possible, dimensions of the microscopic elements are given as: (minimum–) average minus standard deviation – average plus standard deviation (–maximum) of length × (minimum–) average minus standard deviation – average plus standard deviation (–maximum) of width. Spore dimensions do not include the hilar appendix. The width of each basidium was measured at the widest part, and the length was measured from the apex (sterigmata excluded) to the basal septum. The DNA fluorescent dye 4′,6-diamidino-2-phenyl-indoldihydrochloride (DAPI) was used to stain nuclei in spores following Horton (2006). The number of nuclei in spores were then determined using a Leica TCS-SP2 confocal microscope. Samples were excited with 405 nm light and fluorescence was recorded at 440–500 nm. The following abbreviations are used: l = number of lamellulae between each pair of lamellae reaching the stipe; the notation [X, Y, Z] indicates that measurements were made on X randomly selected spores (taken from spore-prints), in Y samples from Z collections; Q = the spore quotient (length/width ratio); Qav = the average spore quotient. Terminology for descriptive terms is according to Vellinga (1988, 2001). Herbarium abbreviations follow Thiers (2019, continuously updated). Author citations follow the Index Fungorum – Authors of Fungal Names (http://www.indexfungorum.org/authorsoffungalnames.htm).

### DNA extraction, PCR amplification and DNA sequencing

Total DNA was extracted from seven dry basidiomes (Tab. 1): two basidiomes (labelled as “a” and “b”) from the same *L.psalion* CAG P.11_9/7.68 collection, one basidiome from the *L.psalion* holotype (WU 5152), two basidiomes from two collections of the new species *L.recondita*, and two basidiomes from two collections of *L.sanguineofracta* Vizzini (TO-HG2916, holotype and TO-HG2917). DNA extraction and PCR amplifications were performed as described by Alvarado et al. (2015). Primers ITS1F and ITS4 (White et al. 1990; Gardes and Bruns 1993) were used for the nrITS region; primers LR0R and LR5 (Vilgalys and Hester 1990) were used for the nrLSU (28S) rDNA, and finally EF1-983F and EF1-1567R (Rehner and Buckley 2005) for the translation elongation factor 1-α (*tef1-α*) gene. Chromatograms were checked searching for putative reading errors, and these were corrected. The PCR products were purified with the Wizard SV Gel and PCR Clean-UP System (Promega) following manufacturer’s instructions and sequenced forward and reverse by MACROGEN Inc. (Seoul, Republic of Korea). Sequences were checked and assembled using Geneious v. 5.3 (Drummond et al. 2010) and submitted to GenBank (http://www.ncbi.nlm.nih.gov/genbank/). Accession numbers are reported in Table 1.

**Table 1.** Taxa, vouchers and GenBank accession numbers used in the molecular analyses. Newly sequenced collections are in bold.

Species	Collection No.	Origin	GenBank accession No.	
			nrITS	nrLSU
* Chamaemyces fracidus *	Th.W. Kuyper 960 (L)	Belgium	AY176343	AY176344
* Cystolepiota cystophora *	MCVE 56163	Italy	GQ141550	–
* Cystolepiota seminuda *	4-X-1989, H.A. Huijser s.n. (herb. Huijser)	The Netherlands	AY176350	–
	MCVE 9247	Italy	JF907983	–
Lepiota aff. grangei	TENN 064380, ECV4063	USA	–	MF797685
* Lepiota acutesquamosa *	DUKE-JJ177	USA	–	U85293
* Lepiota albogranulosa *	LAH. NO. 10152012, Holotype	Pakistan	LK932284	–
	LAH. NO. 9992012	Pakistan	LK932285	–
* Lepiota apatelia *	26-IX-1990, H.A. Huijser (herb. Huijser)	The Netherlands	AY176462	–
	04-X-1991, H.A. Huijser (herb. Huijser)	The Netherlands	GQ203819	–
* Lepiota aspera *	E.C. Vellinga 2233 (L)	The Netherlands	AY176354	–
	GLM 45944	Germany	–	AY207219
* Lepiota bengalensis *	Iqbal 825 GDGM 45684 Holotype	Bangladesh	KU563148	KU563150
	Iqbal 860 Paratype	Bangladesh	KU563149	–
* Lepiota brunneoincarnata *	DB4157	Hungary	–	MK278258
	NL-5409	Hungary	–	MK278260
* Lepiota castanea *	TENN 064371, ECV4016	USA	–	MF797675
	NL-2980	Hungary	–	MK278259
* Lepiota castaneidisca *	E.C. Vellinga 2594 (UC)	USA	AF391055	–
	E.C. Vellinga 2410 (UC)	USA	AF391064	–
	E.C. Vellinga 2805 (UC)	USA	GQ203808	–
	E.C. Vellinga 2756 (UC)	USA	GQ203816	–
Lepiota cf. aspera	MFLU 09-0061	Thailand	–	HM488788
Lepiota cf. cristata	E.C. Vellinga 2515 (UC)	USA	AF391052	–
	E.C. Vellinga 2677 (UCB)	USA	AY176466	–
	E.C. Vellinga 2714 (UC)	USA	GQ203807	–
* Lepiota clypeolaria *	E.C. Vellinga 1683 (L)	Germany	AY176361	–
	TENN 064372, ECV4003	USA	–	MF797684
	VPI-OKM22029	South Korea	–	U85291
	CBS 146.42	Sweden	–	MH867601
* Lepiota coloratipes *	9-X-1991, H.A. Huijser (herb. Huijser)	The Netherlands	AF391066	–
	MCVE 16888	Italy	FJ998406	–
	Zhu L. Yang 4790	China	KC819621	–
	Zhu L. Yang 4951	China	KC819622	–
	SAV F-3212	Spain	KC900376	–
	SAV F-3213, Holotype	Spain	KC900377	–
	NL-5353	Hungary	–	MK278270
* Lepiota cortinarius *	NL-1602	Hungary	–	MK278262
* Lepiota cristata *	22-IX-1993, H.A. Huijser (herb. Huijser)	The Netherlands	AF391042	–
	20-IX-1989, H.A. Huijser (L)	The Netherlands	AF391043	–
	9-VII-1998, Z.L. Yang 2238 (HKAS)	China	AF391044	–
	8-XII-2000, E.C. Vellinga 2611 (UC)	USA	AF391045	–
	30-I-1993, D.E. Desjardin 5658 (SFSU)	USA	AF391050	–
	24-IX-2000, S. Clark (coll. P.B. Matheny 1958) (WTU)	USA	AF391051	–
	AFTOL-ID 1625, ECV 2449 (UC)	USA	–	DQ457685
	E.C. Vellinga 2780 (UC)	USA	GQ203806	–
	E.C. Vellinga 2750 (UC)	USA	GQ203815	–
	DUKE1582	USA	–	U85292
	420526MF0542	China	–	MH141343
	420526MF0550	China	–	MG712361
* Lepiota cristatoides *	5-IX-1996, H.A. Huijser s.n. (herb. Huijser)	The Netherlands	AY176363	–
* Lepiota cystophoroides *	E.C. Vellinga 2142 (L)	France	AF391031	–
* Lepiota erminea *	NL-3095	Hungary	–	MK278263
* Lepiota felina *	VPI-OKM20596	USA	U85330	U85295
	NL-4207	Slovakia	–	MK278264
* Lepiota geogenia *	MEL 2358504	Australia	–	JX179270
	MEL:2358503	Australia	–	JX179271
* Lepiota griseovirens *	MCVE 13747	Italy	FJ998403	–
* Lepiota hymenoderma *	E.C. Vellinga 2017 (L)	The Netherlands	AF391083	–
* Lepiota laevigata *	FP2012-11-02	Hungary	–	MK278266
* Lepiota lilacea *	E.C. Vellinga 2451 (UCB)	USA	AY176379	–
	E. Brown (coll. E.C. Vellinga 1873) (L)	United Kingdom	GQ203820	–
* Lepiota luteophylla *	H.V. Smith 284 (MICH)	USA	AY176475	–
* Lepiota maculans *	TENN 064381	USA	–	HQ832458
* Lepiota mandarina *	HKAS 50028	China	–	KM214816
* Lepiota neophana *	E.C. Vellinga 2602 (UCB)	USA	AY176492	–
	E.C. Vellinga 3947 (UC)	USA	GQ203812	–
	rh24 08/27/07 (ISC)	USA	GQ375546	–
	rh39 08/11/07 (ISC)	USA	GQ375547	–
	E.C. Vellinga ecv3955 (UC)	USA	–	HM488785
* Lepiota ochraceofulva *	E.C. Vellinga 2267) (L)	The Netherlands	AF391032	–
	E.C. Vellinga 2273 (L)	The Netherlands	AY176386	–
* Lepiota ochraceofulva *	NL-2973	Hungary	–	MK278267
* Lepiota ochraceoumbonata *	Murhula Cizungu 39	Gabon	–	MK278268
* Lepiota oreadiformis *	FO 46679	Germany	–	AF291344
* Lepiota phaeoderma *	E.C. Vellinga 3000 (UC)	USA	GQ203810	–
*** Lepiota psalion ***	**WU 5152 Holotype**	**AUSTRIA**	**MG581687**	**MG581699**
***Lepiotapsalion*** basidiome **a**	**CAG P.11_9/7.68**	**Italy**	**MG581688**	–
***Lepiotapsalion*** basidiome **b**	**CAG P.11_9/7.68**	**Italy**	**MG581689**	**MG581700**
*Lepiotapsalion* (*L.recondita*)	15-IX-1999, H.A. Huijser (herb. Huijser) hah6153	The Netherlands	AY176390	–
	3-VIII-1999, H.A. Huijser s.n. (herb. Huijser)	The Netherlands	–	AY176391
	H.A. Huijser (herb. Huijser) hah6177	The Netherlands	GQ203823	–
*Lepiotapsalion* (*L.sinorecondita ad interim*)	HMJAU3799	China	GU199362	GU199355
* Lepiota pseudohelveola *	GLM 45945	Germany	–	AY207220
* Lepiota pyrochroa *	E.C. Vellinga 2006 (L)	The Netherlands	AY176477	–
*** Lepiota recondita ***	**TR gmb 01481, paratype**	**The Netherlands**	**MK508899**	**MK508901**
	**TR gmb 01482, holotype**	**The Netherlands**	**MK508900**	**MK508902**
* Lepiota rhodophylla *	E.C. Vellinga 2610 (UCB)	USA	AY176480	–
*** Lepiota sanguineofracta ***	**TO-HG2916, Holotype**	**Italy**	KF879620	**MG581701**
	**TO-HG2917**	**Italy**	KF879621	**MG581702**
* Lepiota scaberula *	E.C. Vellinga 2307 (UC)	USA	AF391029	–
	E.C. Vellinga 2595 (holotype) (UC)	USA	AF391030	–
	UC1999143	USA	–	MK278271
* Lepiota subcastanea *	HKAS 45633	China	–	KM214817
* Lepiota subgranulosa *	ANGE253 (JBSD, duplicate in MEXU)	The Dominican Republic	KR022007	–
* Lepiota subalba *	E.C. Vellinga 2242 (L)	The Netherlands	AY176489	–
* Lepiota subincarnata *	E.C. Vellinga 2234 (L)	The Netherlands	AY176491	–
	VPI-OKM22153	South Korea	–	U85294
	NL-2022	Hungary	–	MK278273
* Lepiota thiersii *	E.C. Vellinga 2590 (UCB)	USA	AY176485	–
	E.C. Vellinga 2589 (UC)	USA	GQ203817	–
* Lepiota xanthophylla *	TUB 011553	Germany	–	DQ071712
Uncultured Basidiomycota	Environmental sample, man22_soil_G02	USA	GU328508	–

### Sequence alignment, dataset assembly and phylogenetic analysis

Sequences obtained in this study were compared to those available in the GenBank (http://www.ncbi.nlm.nih.gov/) and UNITE (http://unite.ut.ee/) databases by using the Blastn algorithm (Altschul et al. 1990).

**Figure 1.**
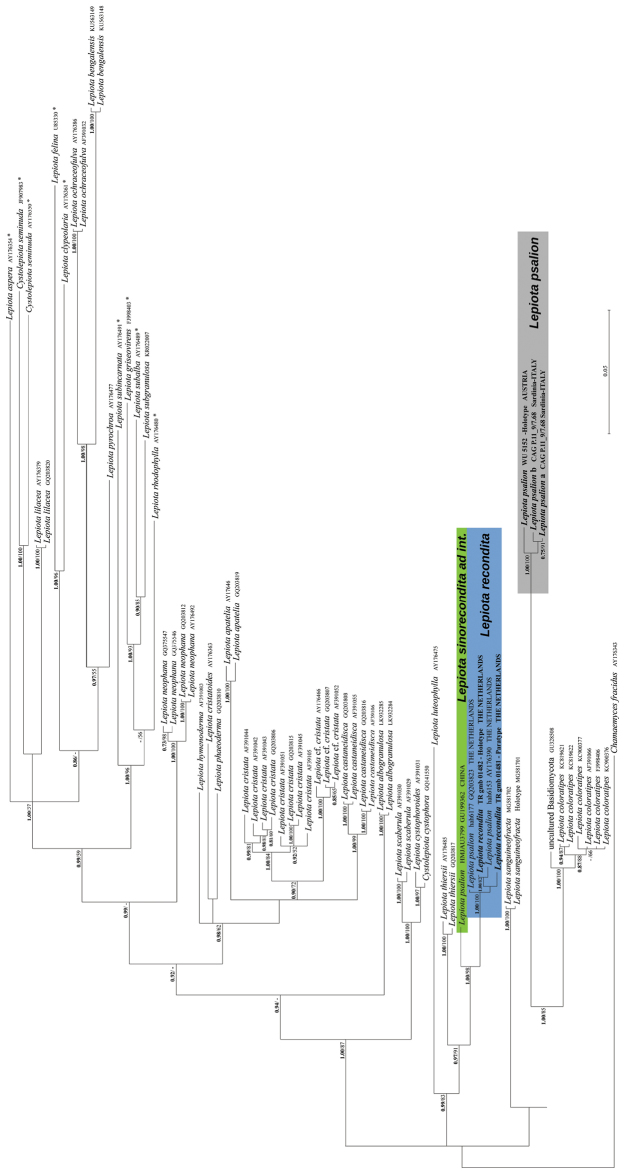
Bayesian phylogram obtained from the general nrITS sequence alignment of *Lepiota* spp. Here there are included *Lepiota* species with a hymeniform pileus covering, eight species representative of the major clades in *Lepiota* (indicated by *), and *Chamaemycesfracidus* as an outgroup taxon. Support values in either the Bayesian (Posterior Probabilities values [BPP]) or Maximum likelihood (ML Bootstrap percentage [MLB]) analyses are indicated. Only BPP values over 0.70 (in bold) and MLB values over 50% are given above clade branches. Newly sequenced collections are in bold.

Based on the BLASTn results (sequences were selected based on the greatest similarity) and outcomes of recent phylogenetic studies incorporating *Lepiota* sequences (Vellinga 2003, 2004, 2010; Vizzini et al. 2014a, b; Justo et al. 2015; Qasim et al. 2015; Hosen et al. 2016) sequences were retrieved from GenBank for the comparative phylogenetic analysis. The nrITS and nrLSU datasets were analysed separately. The combined nrITS/nrLSU phylogeny was not inferred as most *Lepiota* collections in GenBank are not provided with both molecular markers (Table 1). Although *tef1-α* sequences were generated for *L.psalion*, they were not included in phylogenetic analyses because comparable sequences for most *Lepiota* taxa are currently unavailable in public databases, and, in this case, only the Blastn results were provided in the Results. In the nrITS dataset, besides *Lepiota* species with a hymeniform pileus covering, eight species (indicated by an asterisk in Fig. 1) representative of the major clades in *Lepiota* as delimited by Vellinga (2003) were chosen for comparison. The nrLSU dataset consists of all the *Lepiota* s.l. collections determined at species level present in GenBank. Alignments were generated for each nrITS and nrLSU dataset using MAFFT (Katoh et al. 2002) with default conditions for gap openings and gap extension penalties. The two alignments were imported into MEGA v. 6.0 (Tamura et al. 2013) for manual adjustment. The best-fit substitution model for each single alignment was estimated by the Bayesian information criterion (BIC) with jModelTest 2 (Darriba et al. 2012). The GTR + G model was chosen for the nrITS alignment and the TrN+I+G for the nrLSU alignment. The nrITS dataset was partitioned into ITS1, 5.8S and ITS2 subsets. *Chamaemycesfracidus* (AY176343 and AY176344) was used as an outgroup taxon in both the nrITS and nrLSU analyses because it is basal in the Agaricaceae (Vellinga 2004, 2010).

Phylogenetic hypotheses were constructed with Bayesian inference (BI) and Maximum likelihood (ML) criteria. The BI was performed with MrBayes v. 3.2.6 (Ronquist et al. 2012) with one cold and three incrementally heated simultaneous Monte Carlo Markov chains (MCMC) run for 10 million generations, under the selected evolutionary model. Two simultaneous runs were performed independently. Trees were sampled every 1,000 generations, resulting in overall sampling of 10,001 trees per single run; the first 2,500 trees (25%) were discarded as burn-in. For the remaining trees of the two independent runs, a majority rule consensus tree showing all compatible partitions was computed to obtain estimates for Bayesian posterior probabilities (BPP).

**Figure 2.**
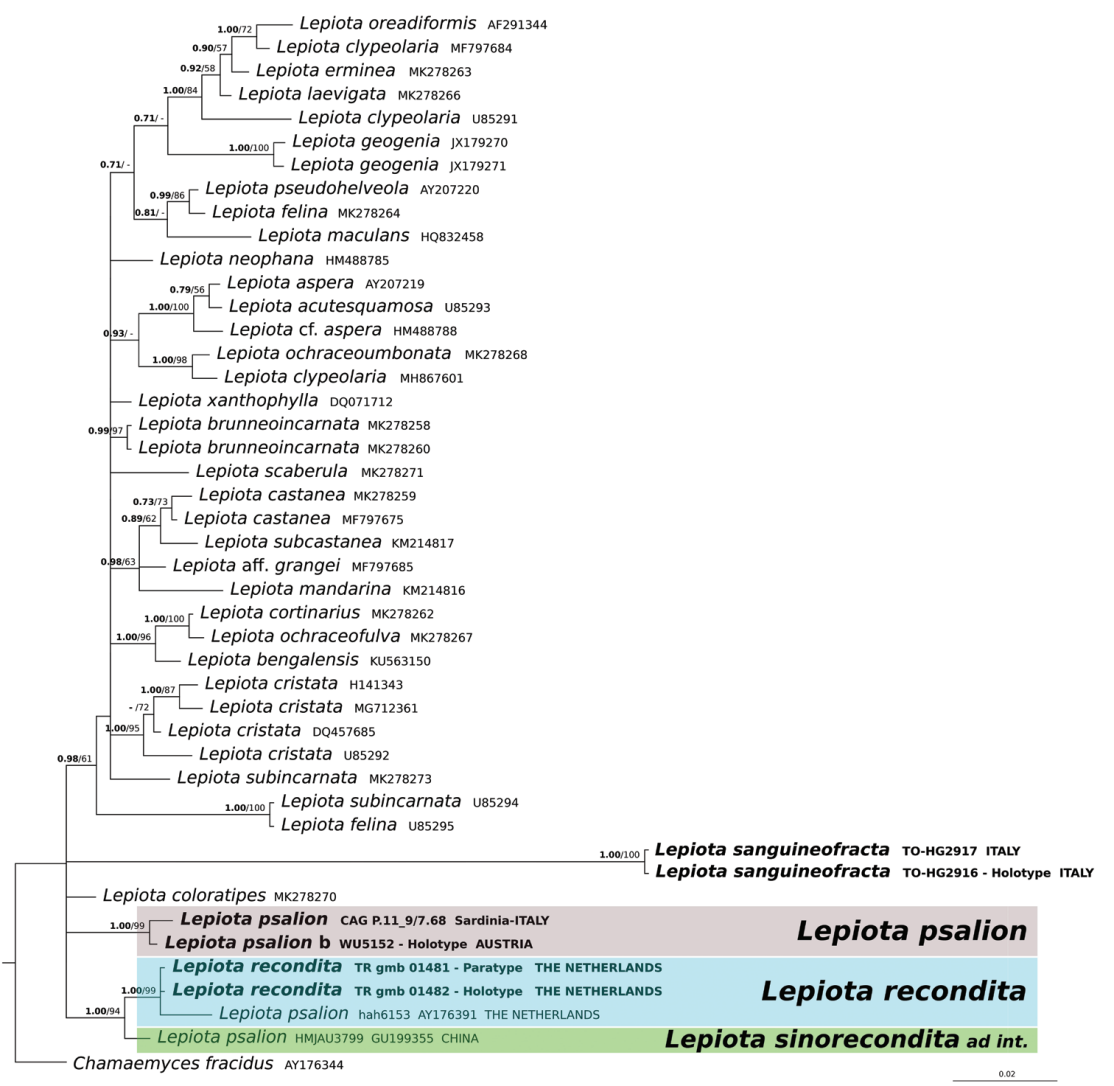
Bayesian phylogram obtained from the general nrLSU sequence alignment of *Lepiota* spp. *Chamaemycesfracidus* was used as an outgroup taxon. Support values in either the Bayesian (Posterior Probabilities values [BPP]) or Maximum likelihood (ML Bootstrap percentage [MLB]) analyses are indicated. Only BPP values over 0.70 (in bold) and MLB values over 50% are given above clade branches. Newly sequenced collections are in bold.

ML estimation was performed with RAxML v. 7.3.2 (Stamatakis 2006), with 1,000 bootstrap replicates (Felsenstein 1985) using the GTRGAMMA algorithm to perform a tree inference and search for a good topology. Support values from bootstrapping runs (MLB) were mapped on the globally best tree using the “-f a” option of RAxML and “-x 12345” as a random seed to invoke the novel rapid bootstrapping algorithm. BI and ML analyses were run on the CIPRES Science Gateway web server (Miller et al. 2010). Only BPP and MLB values over 0.70 and 50%, respectively, are reported in the resulting trees (Figs 1, 2). Pairwise % identity values (P%IV) of the sequences were calculated using MEGA v. 6.0 (Tamura et al. 2013). Alignments and phylogenetic trees are available at TreeBASE (www.treebase.org) under ID S22021.

## Results

### Molecular analysis

The PCR product was 476–729 bp (nrITS) and 894–1128 bp (nrLSU). The nrITS data matrix comprised 68 sequences (including 63 from GenBank). This dataset was 814 bp long and contained 545 (66.9 %) variable sites. The nrLSU data matrix comprised 45 sequences (including 39 from GenBank). This dataset was 953 bp long and contained 335 (35.2%) variable sites.

As both Bayesian and Maximum likelihood analyses produced a consistent topology, only the Bayesian trees with both BPP and MLB values are shown (Figs 1, 2).

In both the nrITS and nrLSU analyses (Figs 1, 2), the sequences of the holotype of *L.psalion* and of the Sardinian collection clustered together in a strongly supported clade (BPP = 1.00, MLB = 100% and BPP = 1.00, MLB = 99%, respectively). The sequences of this clade show a P%IV of 98.9% for the nrITS and of 99.6% for the nrLSU. According to the nrITS analysis, which is based on a larger taxon sampling (Fig. 1), *L.psalion* is sister (BPP = 1.00; MLB = 85%) to *L.coloratipes* Vizzini, J.F. Liang, Jančovičová & Zhu L. Yang. The Blastn results of the *tef1-α* sequences obtained from the two Sardinian specimens of CAG P.11_9/7.68 (MG597229 and MG597230) show an identity value of 83% with *Lepiotaphaeoderma* Vellinga (GQ375549), 81% with *Coniolepiotaspongodes* (Berk. & Broome) Vellinga (HM488881, HM488883 and HM488884) and with *Lepiotaneophana* Morgan (GQ375550 and GQ375551).

Both the nrITS and nrLSU analyses (Figs 1, 2) highlight the presence of sequences in GenBank from Dutch [GQ203823, AY176390 (nrITS), the Netherlands, Limburg province, Valkenburg, Schaelsberg, H.A. Huijser (herb. Huijser), 15-IX-1999, and AY176391 (nrLSU), ibidem, H.A. Huijser (herb. Huijser), 23-VIII-1999] and Chinese collections [GU199362 (nrITS) and GU199355 (nrLSU), China: Jilin province, Changchun, Jinyuetan Park, herb. HMJAU3799] which are named as “*Lepiotapsalion*”, but are clearly distinct from the holotype and the Sardinian collection of *L.psalion*. The Dutch “*Lepiotapsalion*” sequences form a strongly supported clade (BPP = 1.00 and MLB = 100% in the nrITS analysis; BPP = 1.00 and MLB = 99% in the nrLSU analysis) with sequences from the two collections of *L.recondita* (recondita clade). The sequences of this clade show a P%IV of 99.3% for both the nrITS and the nrLSU. The Chinese “*Lepiotapsalion*” is sister (BPP = 1.00 and MLB = 98% in the nrITS analysis; BPP = 1.00 and MLB = 94% in the nrLSU analysis) to the recondita clade.

### Taxonomy

#### Lepiota psalion

Taxon classificationFungiAgaricalesAgaricaceae

Huijser & Vellinga, in Vellinga & Huijser, Belg. J. Bot. 131(2): 203 (1999) [1998]

Figs 3 , 4 , 5 , 6

##### Description.

**Figure 3.**
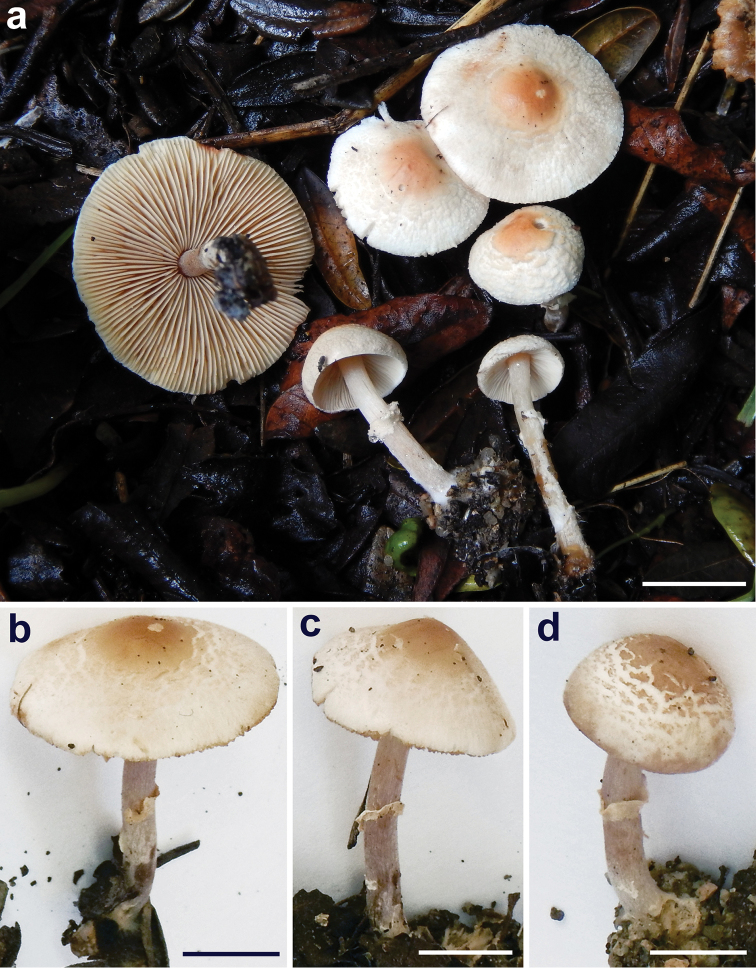
*Lepiotapsalion*. Fresh basidiomes (CAG P.11_9/7.68) **a** Basidiomes in situ **b–d** Details of pileus surface, stipe and annulus. Scale bars: 10 mm (**a**); 5 mm (**b–d**). Photographs by A. Tatti.

Macrocharacters (Fig. 3). *Pileus* 8–36 mm wide, at first slightly obtusely campanulate, hemispherical-trapezoid or broadly conical, later plano-convex to applanate-expanded, subumbonate, with a shallow umbo; not hygrophanous; margin not striated, slightly exceeding the lamellae when young, sinuous-undulate, entire or slightly fringed with age, with minute adhering remnants of partial veil when young; surface dry, at first smooth, later irregularly cracking around centre into concentric non-uplifted squamules; cream to pinkish-light brown at centre [*Vinaceous-buff (Plate XL 17’’’.c-y./d) HTML d3b094 to Orange-Cinnamon (Plate XXIX 13’’.ou-o.) or Ochraceous-Tawny (Plate XV 15’.y-o./i) HTML bc7e4d], paler towards the margin [Pale Cinnamon-Pink (Plate XXIX - 13’’.oy-o./f) HTML e5d6c3 to Pale Smoke-Gray (Plate XLVI 21’’’’.o-y./d) HTML cdc9c6]. *Stipe* 22–33 × 1.5–2 mm, central, cylindrical, usually regular, but sometimes also slightly flexuous, hollow; shiny, at first white, soon becoming pink-brown [Tilleul-Buff (Plate XL - 17’’’.c-y./f), HTML c3b092 to *Drab Gray (Plate XLVI 17’’’.o-y./d) HTML bda599] starting from the base and progressing upward; minutely silky fibrillose along all length; with whitish [Pale pinkish buff (Plate XXIX 17’’.o-y./f) HTML ede2d4], ascending and often incomplete annulus on the upper part of the stipe, sometimes disappearing in age; often with minute white rhizomorphs. *Lamellae* 2–3(4) mm wide, l = 1–3(4), free, crowded, at first white, soon with evident pinkish tints [Cream-Buff (Plate XXX 19’’ .yo-y /d) HTML dfc38c to Clay-Color Plate (XXIX 17’’ .o-y.) HTML ce9b44]; edge finely granulose. *Context* elastic, whitish, pink-brown towards the stipe base; without specific smell and taste. *Spore-print* pale cream.

Microcharacters (Figs 5, 6). *Spores* [700, 6, 2] (2.7–)3.5–4.3(–4.9) × (2.0–)2.6–3.2(–3.9) μm, on average 3.9 × 2.9 μm, Q = (1.03–)1.23–1.49(–1.78), Qav = 1.36, from broadly ellipsoid to ellipsoid, hyaline, thin-walled, smooth, not verruculose in Melzer’s reagent, binucleate, not metachromatic in Cresyl Blue, nonamyloid, non-dextrinoid, cyanophilic in Cotton Blue (Figs 5e, f, 6c). *Basidia* mainly 4-spored, (15.5–)17.1–21(–22.0) × (4.2–)4.7–5.8 (–6.0) μm (*n* = 54), rarely 1- or 2-spored, clavate, hyaline, thin-walled; sterigmata (2.6–) 3.0–4.2 (–4.9) × (0.5–)0.6–1.1(–1.2) μm (*n* = 67) (Fig. 6d). *Lamella edge* sterile. *Cheilocystidia* (10.0–)13.7–21.1 (–26.3) × (4.6–)6.2–8.7(–10.0) μm (*n* = 84), numerous and crowded, hyaline, thin-walled, various in shape, mostly clavate to subutriform, occasionally subfusiform, subcapitulate (Figs 5c, 6b). *Pleurocystidia* absent. *Pileus covering* a (140.7–)153.7–179.1(–201.1) μm (*n* = 16) thick hymeniderm with transition to an epithelium (Figs 5a,b, 6a), with up to 2(or 3) colourless elements on top of each other; terminal elements not tightly packed, (10.4–)18.0–53.6(–62.3) × (3.9–)7.7–19.3(–24.0) μm (*n* = 62), vesiculose, sphaeropedunculate to clavate-pyriform, utriform; slightly thick-walled (walls ca 0.5 μm), with walls embedded in a thin gelatinous matrix; subpellis composed of densely arranged and branching cylindrical hyphae, (21.3–)49.0–108.5(–136.8) × (3.8–)4.5–8.8(–9.7) µm (*n* = 38). *Pileitrama* of cylindrical hyphae, (33.1–)42.1–93.2(–111.8) × (2.7–)4.3–9.8(–14.4) µm (*n* = 45). *Hymenophoral trama* subregular, consisting of cylindrical hyphae (33.8–)36.5–64.4(–83.1) × (6.0–)7.6–15.8(–17.3) µm (*n* = 61). *Stipe covering* consisting of cylindrical hyphae, (23.8–)80.1–214.4(–370.8) × (2.6–)5.4–12.1(–15.4) µm (*n* = 58). *Stipe trama* consisting of cylindrical hyphae, (21.8–)58.5–178.9(–302.7) × (2.5–) 3.3–11.6(–12.5) µm (*n* = 32). *Caulocystidia* absent. *Partial veil* (annulus) composed of cylindrical elements, (21.1–)27.5–52.7(–94.7) × (2.2–)2.9–4.8(–8.5) µm (*n* = 36) with terminal clavate elements, (12.4–)17.9–34.0(–40.3) × (8.4–)10.6–17.7(–19.8) µm (n = 60) (Figs 5d, 6e). *Clamp-connections* present and abundant everywhere.

##### Ecology and distribution.

Gregarious on bare soil, in gardens and parks; so far known only from the type locality (Austria) and Sardinia (Italy).

##### Collections examined.

**Figure 4.**
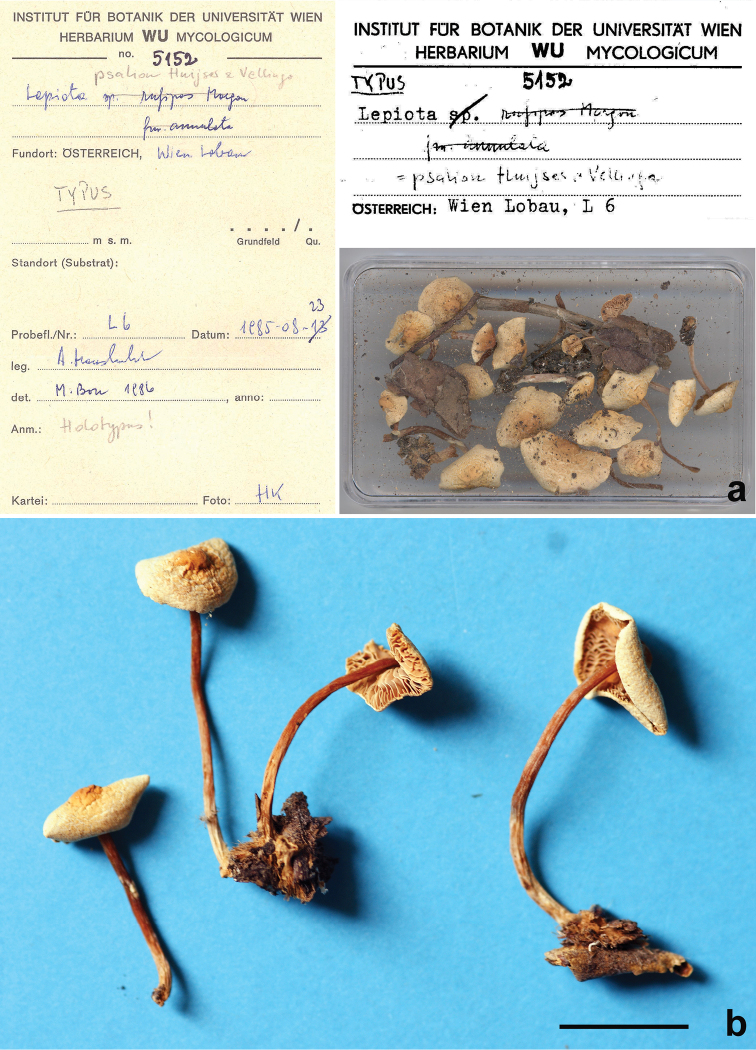
*Lepiotapsalion*. Holotype (WU 5152) **a** Labels and collection **b** Four basidiomes from the collection. Scale bar: 10 mm. Photographs: **a** by W. Till; **b** by A. Vizzini.

Italy, Sardinia, Cagliari, Botanical Garden, 6 basidiomes growing among the *Searsia*/*Rhus* sp. litter, calcareous soil, 17 January 2017, Alessia Tatti and Giacomo Calvia (CAG P.11_9/7.68). Austria, Wien-Lobau, N. Uferhaus, 23 August 1985, Anton Hausknecht (WU 5152, holotype) (Fig. 4).

**Figure 5.**
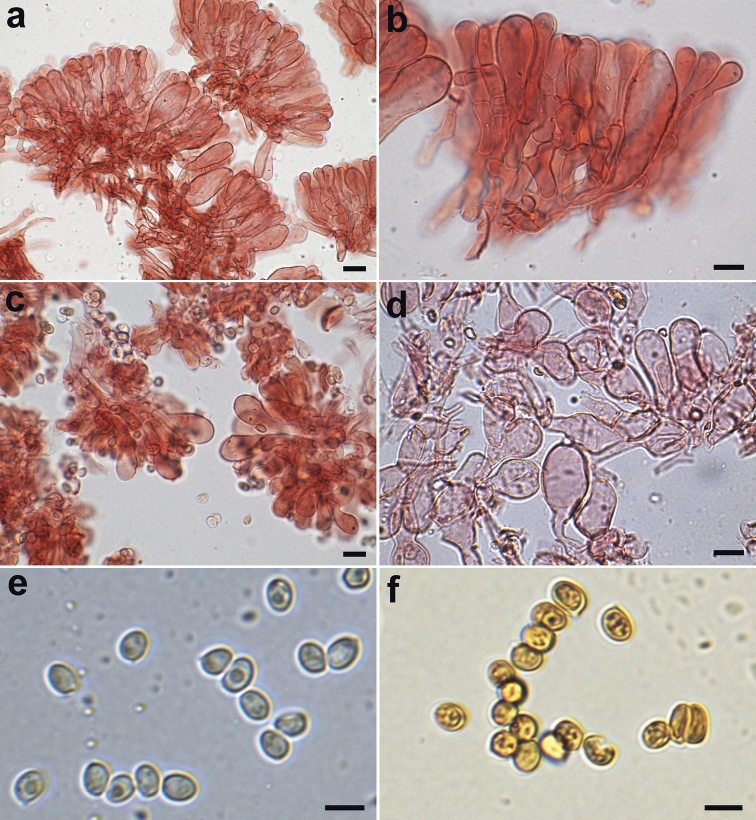
*Lepiotapsalion*. Microscopic features (CAG P.11_9/7.68) **a–b** Elements of the pileus covering **c** Cheilocystidia **d** Elements of the annulus **e–f** Spores. **a–d** in ammoniacal Congo red **e** in 5% KOH **f** in Melzer’s reagent. Scale bars: 10 μm (**a–d**); 5 μm (**e–f**). Photographs by A. Tatti.

**Figure 6.**
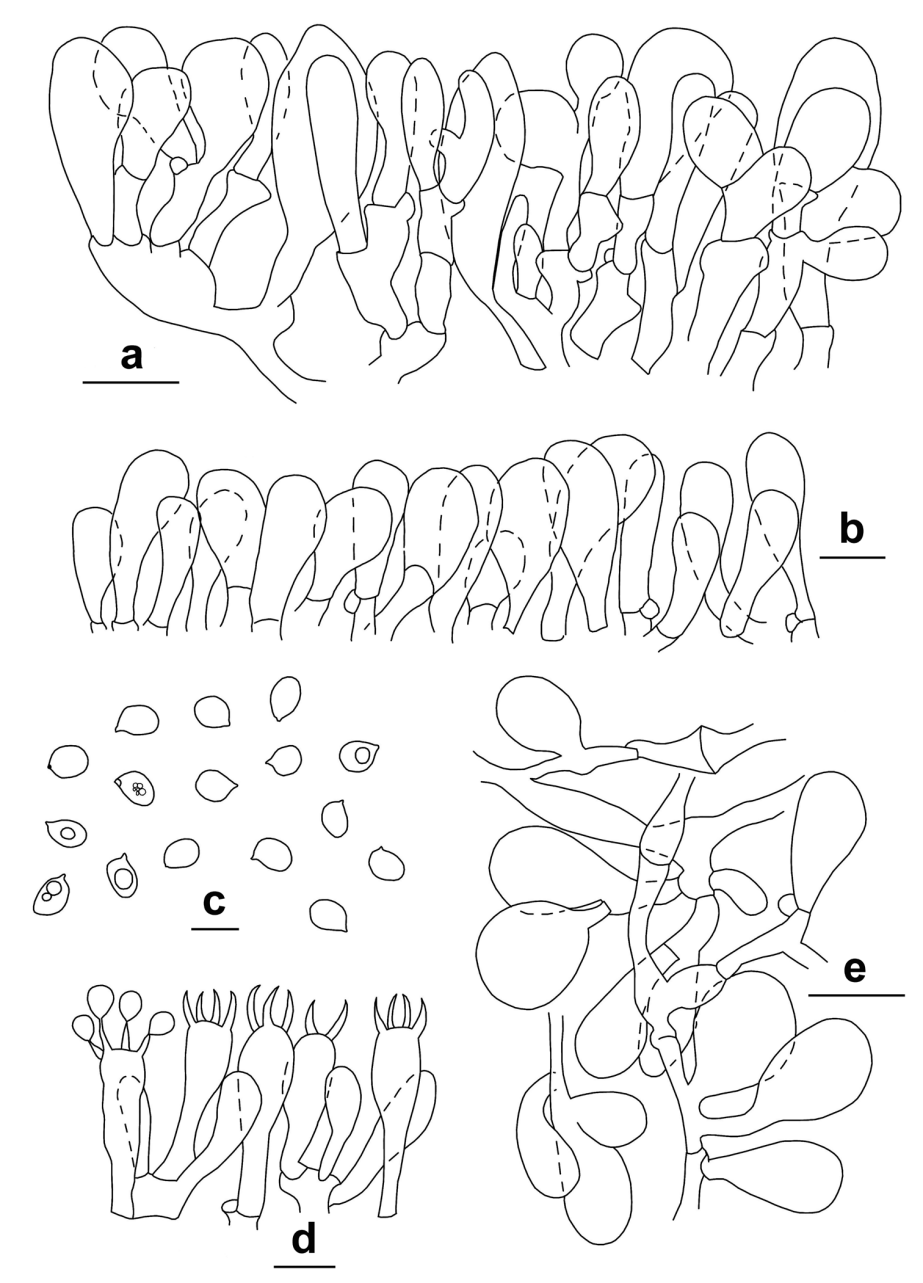
*Lepiotapsalion*. Microscopic features (CAG P.11_9/7.68) **a** Elements of the pileus covering **b** Cheilocystidia **c** Spores **d** Basidia **e** Elements of the annulus. Scale bars: 20 μm (**a, e**); 10 μm (**b, d**); 5 μm (**c**). Drawings by A. Tatti.

#### Lepiota recondita

Taxon classificationFungiAgaricalesAgaricaceae

Tatti, Huijser & Vizzini sp. nov.

MycoBank No: MB 829963

Figs 7 , 8 , 9

##### Holotype.

The Netherlands, prov. Limburg, Valkenburg, Schaelsberg, 02 September 2004, Henk A. Huijser (TR gmb 01482).

##### Etymology.

From the Latin “reconditus”, meaning hidden, forgotten, which refers to its resemblance with *L.psalion* with which it was confused.

##### Diagnosis.

It is distinguished from *Lepiotapsalion* by larger spores (3.7–)4.4–5.4(–5.9) × (2.4–)2.9–3.6(–4.3) μm, versiform cheilocystidia and different nrITS and nrLSU sequences.

##### Description.

**Figure 7.**
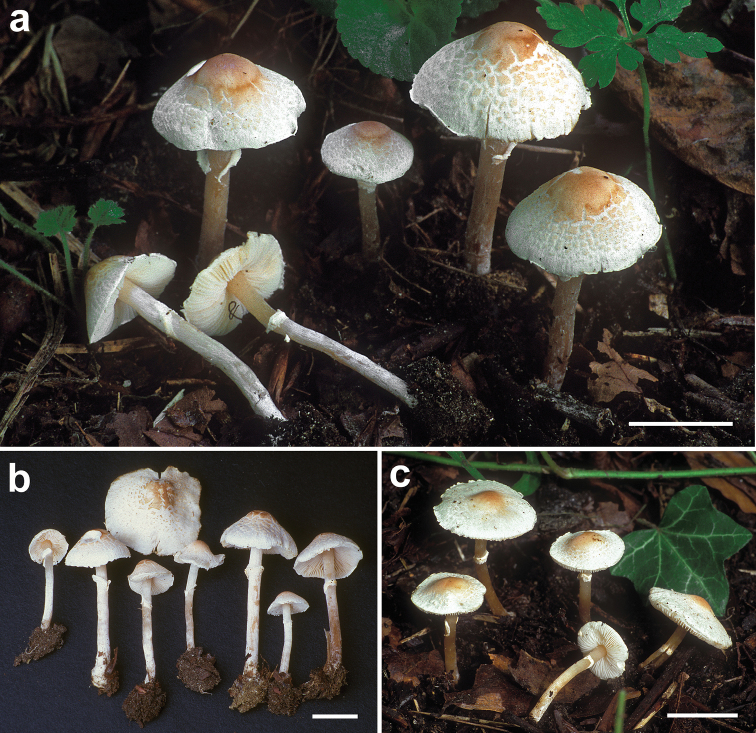
*Lepiotarecondita*. Fresh basidiomes **a–b** (TR gmb 01482, holotype) **c** (TR gmb 01481, paratype). Scale bars= 10 mm. Photographs by H.A. Huijser.

Macrocharacters (Fig. 7). *Pileus* 9–26 mm wide, at first slightly obtusely campanulate, hemispherical-trapezoid or broadly conical, later plano-convex to applanate-expanded, subumbonate, with a shallow umbo; not hygrophanous; margin not striated, slightly exceeding the lamellae when young, sinuous-undulate, entire or slightly fringed with age, with minute adhering remnants of partial veil when young; surface dry, at first smooth, later irregularly cracking around centre into concentric non-uplifted squamules; pinkish-light brown at centre from [Light Pinkish Cinnamon (Plate XXIX, 15’’.Y-O./d) HTML f19b5f] to [Mikado brown (Plate XXIX 13’’.OY-O./i), HTML 9f5425] or [Sayal Brown (Plate XXIX, 15’’.Y-O./i) HTML bc662d], paler towards the margin: [Capucine Bluff (Plate III, 13.OY-O./f) HTML fee6cc] or [Orange Pink (Plate II, 11.ORANGE/f) HTML ecc8a3]. *Stipe* 26–47 × 1.5–3 mm, central, cylindrical, at first white, becoming pink-brown with manipulation [Pinkish Cinnamon (Plate XXIX, 15’’.Y-O./b) HTML e1934f]; minutely silky fibrillose along all length; with whitish, ascending and often incomplete annulus on the upper part of the stipe, sometimes disappearing in age; often with minute white rhizomorphs. *Lamellae* free, crowded, l = 1–3, at first white, soon with evident yellowish tints [Catrige Buff (Plate XXX 19’’ .yo-y /f) HTML cdaf68] becoming [Honey Yellow (Plate XXX 19’’.YO-Y) HTML de9e42] when dry. *Context* elastic, whitish, smell weak, *Lepiotacristata*-like, taste not recorded. *Spore-print* whitish.

**Figure 8.**
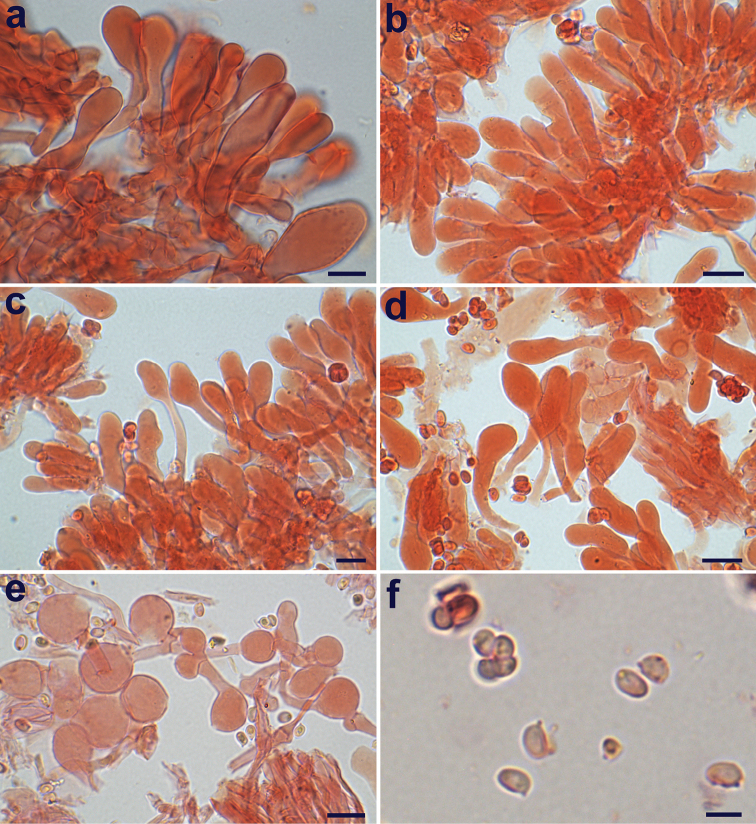
*Lepiotarecondita*. Microscopic features (in ammoniacal Congo red, TR gmb 01482, holotype) **a** Elements of the pileus covering **b–d** Cheilocystidia **e** Elements of the annulus **f** Spores. Scale bars: 10 μm (**a–e**); 5 μm (**f**). Photographs by A. Tatti.

**Figure 9.**
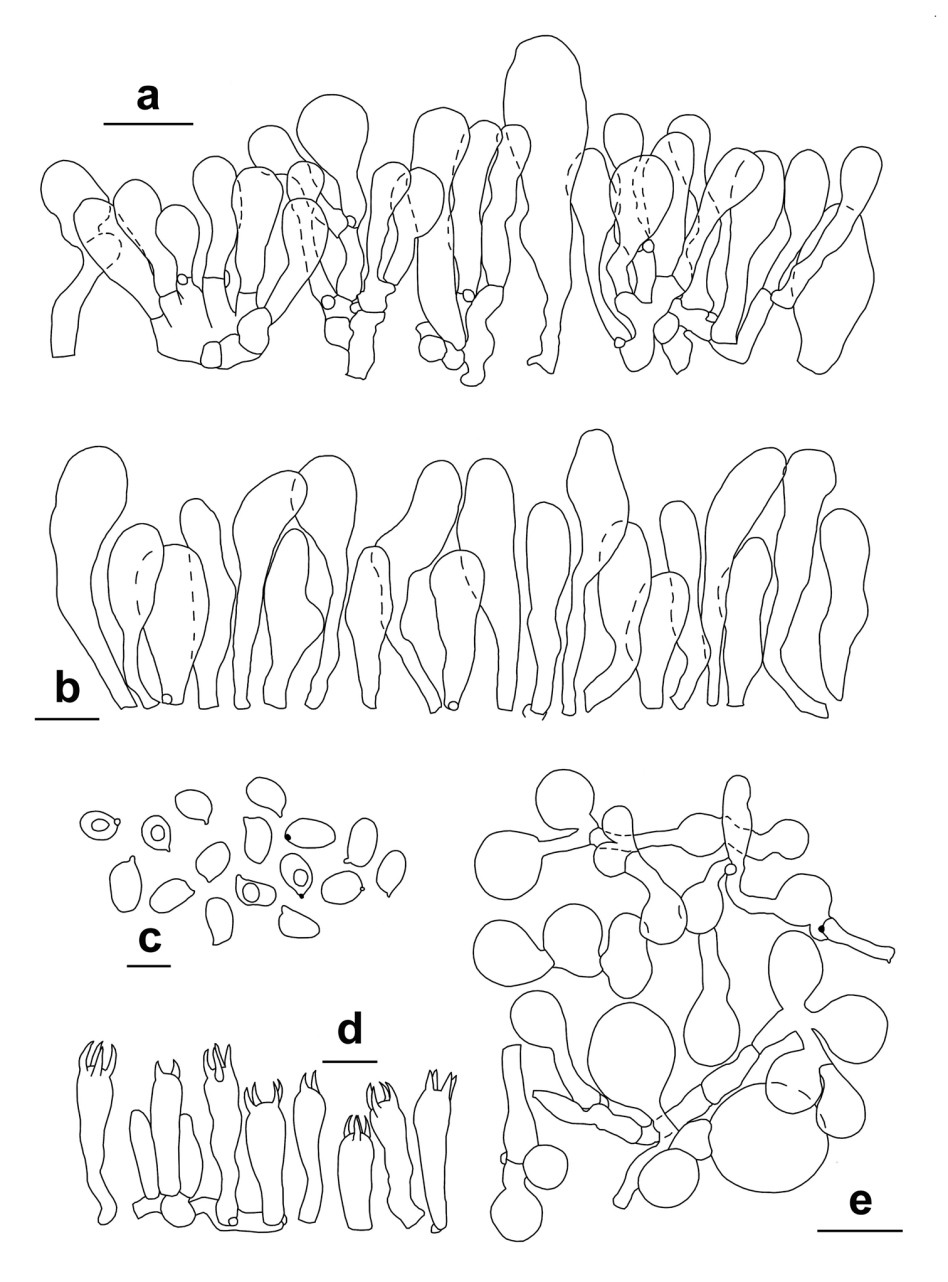
*Lepiotarecondita*. Microscopic features (TR gmb 01482, holotype) **a** Elements of the pileus covering **b** Cheilocystidia **c** Spores **d** Basidia **e** Elements of the annulus. Scale bars: 20 μm (**a, e**); 10 μm (**b, d**); 5 μm (**c**). Drawings by A. Tatti.

Microcharacters (Figs 8, 9). *Spores* [350, 6, 2] (3.7–)4.4–5.4(–5.9) × (2.4–)2.9–3.6(–4.3) μm, on average 4.8 × 3.3 μm, Q = (1.1–)1.3–1.7(–2.0), Qav = 1.5, from subglobose to oblong, mainly ellipsoid, hyaline, thin-walled, smooth, not verruculose in Melzer’s reagent, binucleate, not metachromatic in Cresyl Blue, nonamyloid, non-dextrinoid, cyanophilic in Cotton Blue (Figs 8f, 9c). *Basidia* mainly 4-spored, (15.8–)17.4–25.4(–28.6) × (5.7–)6–7.3(–8.8) μm (*n* = 60), sometimes 1–2-spored, clavate, hyaline, thin-walled (Fig. 9d); sterigmata (1.9–)2.4–4.2(–4.8) × (0.4–)0.6–1.2(–1.5) µm (*n* = 70). *Lamella edge* sterile. *Cheilocystidia* (20.1–)25.4–44(–50.0) × (3.2–)7.2–10.4(–12.0) µm (*n* = 66), numerous and crowded, hyaline, thin-walled, various in shape, mostly clavate, cylindrical-clavate, sphaeropedunculate to submoniliform, occasionally pyriform, cylindrical (Figs 8b–d, 9b). *Pleurocystidia* absent. *Pileus covering* hymenidermic: terminal elements not tightly packed, (17–)24.7–51.1(–59.6) × (8.1–)10–14(–27.3) μm (*n* = 70), vesiculose, sphaeropedunculate to clavate-pyriform (Figs 8a, 9a); slightly thick-walled (walls ca 0.5 μm), with walls embedded in a thin gelatinous matrix; subpellis composed of densely arranged and branching cylindrical hyphae, (40.6–)47.0–118.3(–156.2) × (5.8–)7.6–16.2(–17.1) µm (*n* = 20) and containing scattered ramified oleiferous hyphae, (1.5–)1.8–5.3(–8.0) µm wide (*n* = 30). *Hymenophoral trama* subregular, consisting of ovate hyphae (20.9–)21.1–40.3(–42) × (7–)9.6–13(–14.5) µm (*n* = 12). *Stipe covering* and *trama* indistinguishable, consisting of cylindrical hyphae, (55.3–) 67.0–165.7 (–213.0) × (5.5–)7.6–15.0(–21.0) µm. *Caulocystidia* absent. *Partial veil* (annulus) composed of cylindrical elements, (7.2–)22.3–59(–70.0) × (2.0–)2.5–4.2(–4.7) µm (*n* = 20) with terminal clavate elements, (10.1–)12.4–26.7(–38.1) × (7.0–)9.5–16.7(–28.4) µm (*n* = 40) (Figs 8e, 9e). *Clamp-connections* present and abundant everywhere.

##### Ecology and distribution.

Gregarious on rich in nutrients and lime (marl) bare soil, in a mixed deciduous forest; so far known only from the type locality.

##### Collections examined.

The Netherlands, Limburg province, Valkenburg, Schaelsberg, man-made (anthropized) hilly grove with mainly deciduous trees (*Quercus*, *Fagus*, *Corylus*, *Fraxinus*, *Robinia*, *Prunus*, *Sambucus*), together with *Lepiotatomentella*, *L.poliochloodes*, *Melanophyllumeyrei*, and *Limacellaochraceolutea*, 22 September 2001, Henk A. Huijser (TR gmb 01481, paratype); *ibidem*, 02 September 2004, Henk A. Huijser (TR gmb 01482, holotype).

#### Lepiota sinorecondita

Taxon classificationFungiAgaricalesAgaricaceae

ad interim

Fig. 10

##### Description.

The specific epithet is a combination of Medieval Latin “sino” (which means Chinese) and “recondita”, referring to the strong affinity of the Chinese taxon to the European *L.recondita*.

**Figure 10.**
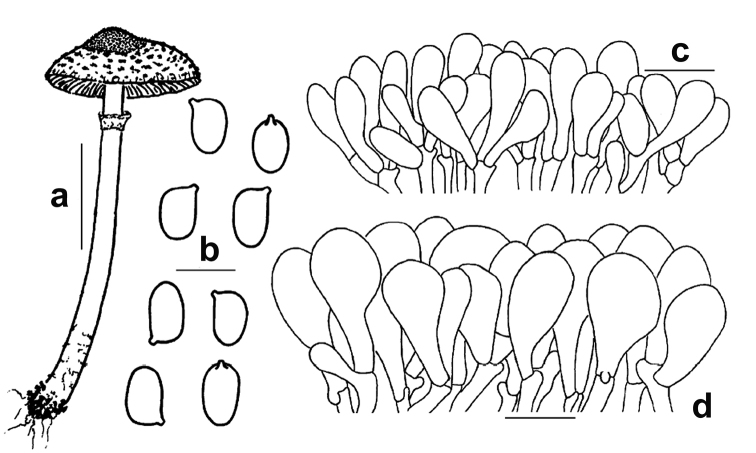
*Lepiotasinorecondita* (HMJAU 3799) **a** Basidiome **b** Spores **c** Cheilocystidia **d** Elements of the pileus covering. Scale bars: 10 mm (**a**); 5 μm (**b**); 20 μm (**c–d**). Drawings by J.F. Liang.

*Basidiomata* small (Fig. 10a). *Pileus* 9–17 mm wide, expanding to convex with obtuse umbo; at centre on umbo smooth, dark yellowish brown to dark brown, around umbo split up into pale brown concentrically arranged patches on dirty white to cream background, paler and smaller towards margin. *Stipe* 35–37 × 1–4 mm, subcylindrical or attenuate, slightly inflated at base; hollow, dirty white and glabrous at the apical part, surface whitish, covered white, tomentose at lower part, with white mycelial cords at base; annulus membranous, superior, whitish on upper surface, with small yellowish brown to brownish squamules on lower whitish surface. *Lamellae* free, cream, yellow to brown when dry, crowded with lamellulae, edge wavy.

*Spores* [60,3,1] (4.0–)4.5–5.5 × 2.5–3.0(–3.5) µm, Q = 1.50–1.80(–1.83), Qav = 1.64 (Fig. 10b), ellipsoid to oblong in side and front view, without suprahilar depression, sometimes with straight adaxial side; hyaline, smooth, non-dextrinoid, congophilous but very weakly, slightly reddish purple in Cresyl Blue. Basidia 17–22 × 5–6 μm, narrowly clavate or subcylindrical, 4-spored. *Lamella edge* sterile. *Cheilocystidia* 21–40 × 6–13 μm, clavate to narrowly clavate, rarely broadly clavate, colourless, hyaline, thin-walled (Fig. 10c). *Pleurocystidia* absent. *Pileus covering* a hymeniderm made up of broadly clavate, clavate to obpyriform terminal elements, 18–50 × 10–20 μm, with pale yellowish brown intracellular pigment (Fig. 10d). *Clamp-connections* present in all tissues.

##### Collection examined.

China, Jilin Province, Changchun City, Jinyuetan Park, 7 July 2005, Wang Jianrui (HMJAU 3799).

##### Ecology and distribution.

Solitary, terrestrial, on the ground in a larch forest in summer and autumn. So far known only from China.

### Discussion

#### Distinguishing characters of *L.psalion* and allied species

The morphological differences among the *Lepiota* species with hymeniform pileus covering are often subtle (Vellinga and Huijser 1999; Vellinga 2010), but nrlTS sequence data support the morphologically recognized species (Vellinga 2010; Vizzini et al. 2014a, b; Justo et al. 2015; Qasim et al. 2015; Hosen et al. 2016).

*Lepiotapsalion* is distinguished by having a non-smooth pileus with concentric non-uplifted squamules, a distinct annulus, and mostly clavate cheilocystidia (Vellinga and Huijser 1999; Vellinga 2001; our observations). The annulus is quite evanescent (Fig. 3) mainly because it is predominantly composed of inflated elements (Figs 5d, 6e).

Lepiota “cf. rufipesf.phaeophylla” sensu Winterhoff and Bon (1994) and *L.rufipes* sensu Babos (1974), Wasser (1980), and Krieglsteiner (1991), all with a distinct annulus, are probably referable to *L.psalion* (Vellinga and Huijser 1999; Vellinga 2001), but see below.

The phylogenetically closest species are *L.coloratipes* (= *L.rufipes* ss. Auct. europ. non ss. orig.) and *L.sanguineofracta* (Fig. 1). *Lepiotacoloratipes* differs from *L.psalion* in having a usually smooth pileus surface, a very evanescent partial veil not forming an annulus but leaving fibrillose remnants on stipe surface, a stipe with reddish tinges at base, the presence of oil droplets in all tissues (including spore surface), the hymeniform pileus covering consisting of very tightly arranged clavate to sphaeropedunculate elements, the presence of uninucleate spores which are often verruculose in Melzer’s reagent, versiform cheilocystidia (mostly lageniform or lecythiform), and the presence of caulocystidia (Bon 1981, 1993; Candusso and Lanzoni 1990; Vellinga and Huijser 1999; Vellinga 2001; Vizzini et al. 2014b). *Lepiotasanguineofracta*, recently described from Italy, is characterized by a micaceous but not squamulose pileus surface with distinct green tinges when mature, a fugacious partial veil not forming an annulus, a stipe with reddish tinges towards the base, the context smelling of dried rose petals, basidiome surfaces and context strongly reddening on handling, binucleate spores, and versiform cheilocystidia (clavate to subutriform, subfusiform) (Vizzini et al. 2014a).

The other morphologically allied species of *Lepiota* with a hymeniform pileus covering, ellipsoid spores, and a well-formed annulus, phylogenetically far from *L.psalion* (Figs 1, 2), show distinctive morphological traits: *L.apatelia* Vellinga & Huijser, *L.cristatoides* Einhell. (both from Europe), and *L.thiersii* Sundb. (from western North America) have no cheilocystidia (Einhellinger 1973; Sundberg 1989; Vellinga and Huijser 1999; Vellinga 2001, 2010; Hausknecht and Pidlich-Aigener 2005; Kosakyan et al. 2008; Mertens 2010; Gierczyk et al. 2011). *Lepiotaneophana* (including var. europaea Bizio & Migl. and f. papillata Migl. & L. Perrone) shows a smooth pileus surface with a buff to dark-brown and umbonate centre, very rare clamp-connections in the pileus trama and no cheilocystidia (Anonymous 1992; Bizio et al. 1993; Vellinga and Huijser 1999; Vellinga 2010). Finally, pale collections of *L.lilacea* Bres. are distinguished by whitish lamellae, an annulus with lilac-brown tinges on the lower part and margin, and metachromatic (in Cresyl Blue) up to 6 µm long spores (Bon 1981, 1993; Migliozzi and Clericuzio 1989; Candusso and Lanzoni 1990; Vellinga 2001).

### The *Lepiotapsalion* complex

*Lepiotapsalion* was established by Vellinga and Huijser (1999) based on an Austrian collection made by A. Hausknecht on 23 August 1985 (WU 5152) and determined by M. Bon as L.rufipesfo.annulata ined. (Fig. 4a). The extended description they provided is heterogeneous: the macromorphology was taken from Krieglsteiner (1991) who described a German collection as *L.rufipes*, collection considered by Vellinga and Huijser as *L.psalion*, while the micromorphology was based on the analysis of the holotype made by the same Dutch mycologists. NrITS and nrLSU sequences later deposited in GenBank as *L.psalion* were generated by Vellinga (2004, 2010) not from the holotype, but from three Dutch collections (vouchers 23-VIII-1999, 15-IX-1999, and hah6177, H.A. Huijser, herb. Huijser).

When the Sardinian specimens were collected, they were morphologically attributed to *L.psalion*, but when they were sequenced to obtain molecular evidence, they did not cluster either with the Dutch collections or with a collection named *L.psalion* from China (herb. HMJAU3799; Liang et al. 2011) (tree not shown). Consequently, we decided to request the holotype collection from WU and sequenced it. Phylogenetic analyses highlighted that Sardinian collection and the holotype are conspecific (Figs 1, 2) and sister to *L.coloratipes* (Fig. 1). Molecular data so confirm *L.psalion* as independent species in the genus *Lepiota*; Dutch and Chinese collections are two distinct and yet undescribed new species, phylogenetically close (BPP = 0.97; MLB = 91%) to *L.thiersii* (Fig. 1). Unfortunately, the collections of the Dutch taxon whose sequences are deposited in GenBank were subsequently lost (Vellinga, pers. comm.) but, based on two newly sequenced additional collections from the same original area of the Dutch taxon, the new species *L.recondita* is here described. As only one collection (consisting of three basidiomes) is available for the Chinese taxon, it was decided to propose it only as an *ad interim* species. Further collections will be necessary to describe it as a new species.

*Lepiotapsalion*, *L.recondita*, *L.* “*sinorecondita*”, *L.apatelia*, and *L.thiersii* constitute a homogeneous morphology-based but not monophyletic group, here named the “*L.psalion* complex”, which is circumscribed by a set of shared characters: a pileus surface breaking into small squamules, well-formed white partial veil (usually forming an annulus, but see *L.apatelia*), hymeniform pileus covering, and ellipsoid spores.

An identification key for the taxa belonging to this complex is proposed below.

### Key to the species of the *Lepiotapsalion* complex

**Table:** 

1	Cheilocystidia absent	**2**
–	Cheilocystidia present	**3**
2	Smell farinaceous, annulus often adhering to pileus margin (as velar remnants), spores weakly dextrinoid	***L.apatelia* (Europe)**
–	Smell *L.cristata*-like, annulus usually ascending on stipe, spores non-dextrinoid	***L.thiersii* (North America)**
3	Spores ellipsoid, on average = 3.9 μm long, Qav = 1.36	***L.psalion* (Europe)**
–	Spores ellipsoid to oblong, on average > 4.0 μm long, Qav > 1.4	**4**
4	Cheilocystidia versiform, spores ellipsoid, Qav = 1.5, annulus entirely smooth	***L.recondita* (Europe)**
–	Cheilocystidia mainly clavate, spores oblong, Qav = 1.64, annulus covered by minute yellowish brown squamules on lower surface	***L.sinorecondita ad int.* (China)**

## Supplementary Material

XML Treatment for Lepiota psalion

XML Treatment for Lepiota recondita

XML Treatment for Lepiota sinorecondita
